# A National Pediatric Cohort Study on In‐Hospital Cardiac Arrest in Sweden

**DOI:** 10.1111/aas.70346

**Published:** 2026-09-27

**Authors:** Hannah Fovaeus, Johan Holmen, Zacharias Mandalenakis, Matilda Frisk Torell, Mattias Molin, Araz Rawshani, Albert Gyllencreutz Castellheim

**Affiliations:** ^1^ Department of Anesthesiology and Intensive Care Medicine, Institute of Clinical Sciences, Sahlgrenska Academy University of Gothenburg Gothenburg Sweden; ^2^ Department of Pediatrics Sahlgrenska University Hospital Gothenburg Sweden; ^3^ Department of Pediatric Anesthesiology and Intensive Care Sahlgrenska University Hospital Gothenburg Sweden; ^4^ Department of Molecular and Clinical Medicine, Sahlgrenska Academy University of Gothenburg Gothenburg Sweden; ^5^ Department of Medicine, Adult Congenital Unit Sahlgrenska University Hospital Gothenburg Sweden; ^6^ Department of Cardiology Sahlgrenska University Hospital Gothenburg Sweden; ^7^ Statistiska Konsultgruppen Gothenburg Sweden

## Abstract

**Background:**

National data on pediatric in‐hospital cardiac arrest (pIHCA) are limited, and cohorts from highly specialized pediatric centers may not reflect the broader hospital population. We aimed to describe pIHCA reported across Swedish hospitals, with particular focus on hospitals outside the two national centers for highly specialized pediatric cardiac care.

**Methods:**

This retrospective observational registry study included patients aged 0–18 years with pIHCA reported to the Swedish Registry for Cardiopulmonary Resuscitation between January 1, 2005 and September 25, 2025. Cases from the two highly specialized centers were analyzed separately and as part of the complete national registry‐reported cohort. The primary outcome was 30‐day survival; secondary outcomes included return of spontaneous circulation (ROSC) and 1‐year survival.

**Results:**

Among 525 pIHCA events, 344 occurred outside the two highly specialized centers and 181 at these centers. In the broader Swedish hospital population, 31.7% of arrests occurred on hospital wards, 26.2% in emergency departments, and 22.7% in intensive care units. CPR was initiated immediately in 81.4% of events. Forty‐nine percent survived to 30 days, and 44% to 1 year. At the two highly specialized centers, infants, congenital heart disease, and arrests in intensive care units were substantially more common, and 30‐day survival was 70%.

**Conclusion:**

Patient characteristics, arrest location, and survival differed substantially between the broader Swedish hospital population and the two highly specialized centers. These findings highlight the heterogeneity of pIHCA across hospital settings and the importance of considering hospital type and case mix when interpreting pIHCA data.

**Editorial Comment:**

Pediatric patient in‐hospital cardiac arrest is uncommon, though they registered in a national database in Sweden. This analysis presents 20 years of experience and follow‐up for these cases describing associated factors.

## 1 Introduction

The systematic collection of data on pediatric in‐hospital cardiac arrest (pIHCA) remains limited in many countries, despite increasing recognition of the importance of benchmarking resuscitation practices and outcomes [1]. pIHCA is an uncommon clinical event that carries a considerable risk of morbidity and mortality. Since 2005, the Swedish Registry for Cardiopulmonary Resuscitation (SRCR) has collected data on in‐hospital cardiac arrests (IHCA) where cardiopulmonary resuscitation (CPR) has been initiated [2]. To our knowledge, data from the complete national pediatric in‐hospital cohort have not previously been published.

Over the last two decades, outcomes following pIHCA have improved substantially. Earlier reports describe survival to hospital discharge rates of approximately 25% [3], whereas more contemporary studies report return of spontaneous circulation (ROSC) in more than 80% of cases and markedly improved survival, although mortality before hospital discharge remains considerable [4, 5]. Survival is strongly influenced by the underlying etiology of the arrest. Children with cardiac arrest secondary to sepsis have been reported to experience poorer outcomes than those with other causes of arrest [6], whereas patients presenting with bradycardia and poor perfusion, particularly in intensive care settings, were associated with better survival [7].

Compared with pediatric out‐of‐hospital cardiac arrest (pOHCA), the in‐hospital cohort represents a different clinical entity. While pOHCA is frequently associated with sudden and often non‐medical events such as drowning, sudden infant death syndrome (SIDS), trauma, suicide, or overdose [8], pIHCA is more commonly preceded by progressive respiratory failure and circulatory shock. In contrast to the sudden cardiac etiologies frequently observed in adults, pediatric arrests are often preceded by a period of physiological deterioration, providing an opportunity for recognition and intervention before cardiac arrest occurs [9, 10, 11]. Consequently, considerable effort has been directed toward early identification of clinical deterioration; for example, through rapid response systems and pediatric early warning scores (PEWS). Although implementation has been associated with a reduction in clinical deterioration events, evidence for improved survival remains inconclusive [12].

Another characteristic of pIHCA is its frequent occurrence in highly monitored environments. Several studies have reported that the majority of pediatric arrests occur in intensive care units [13], where many patients already receive advanced respiratory and circulatory support. This contrasts with adult populations, in which arrests are more evenly distributed across hospital wards and survival remains lower overall. Differences in patient characteristics, arrest etiology, and clinical environment suggest that findings from adult IHCA populations cannot readily be extrapolated to children [4, 14].

Despite international knowledge regarding pIHCA, national population‐based data remain limited. Sweden has maintained a national cardiac arrest registry for more than three decades. However, pediatric‐specific variables were not introduced in the SRCR until 2018 [15], limiting the registry's ability to fully capture pediatric‐specific aspects of cardiac arrest and resuscitation. Furthermore, participation among pediatric referral centers has varied over time. As a result, the epidemiology and outcomes of pIHCA in Sweden remain incompletely described.

The primary aim of this study was to describe the epidemiology, patient characteristics, arrest circumstances, and outcomes of pIHCA reported from Swedish hospitals, with particular focus on the population treated outside the two national centers for highly specialized pediatric cardiac care. By examining these centers separately and as part of the complete national registry‐reported cohort, we also aimed to characterize differences across hospital settings and provide a more comprehensive description of pIHCA in Sweden.

## 2 Methods

### 2.1 Study Design and Population

This retrospective observational study used data from the SRCR. The study included pediatric patients aged 0–18 years with a registered IHCA from January 1, 2005 to September 25, 2025. Age was defined at the time of the cardiac arrest. To provide a comprehensive description of pIHCA across Swedish hospital settings, cases from Sweden's two national centers for highly specialized pediatric cardiology and cardiac surgery: Queen Silvia Children's Hospital (Drottning Silvias Barnsjukhus; DSBS) and Skåne University Hospital/Lund, Children's hospital (Skånes universitetssjukhus in Lund; SUS/L) were analyzed both separately and as part of the complete national registry‐reported cohort. Data from DSBS have previously been described in detail [16]. Separate analyses of these two centers were performed because they represent highly specialized tertiary referral settings and contribute substantially to the pediatric cardiac arrest registry population. Their patient population and case mix may therefore differ from those of other Swedish hospitals and may influence the characteristics and outcomes observed in the national registry‐reported cohort. By presenting results both with the two centers included in the complete cohort and with their data considered separately, we aimed to provide a more comprehensive and nuanced description of pIHCA in Sweden.

### 2.2 SRCR

The SRCR was established in 1990 for out‐of‐hospital cardiac arrest and was expanded in 2005 to include IHCA. It was among the first national registries to systematically collect data on IHCA [17] and remains one of the few nationwide IHCA registries in Europe [1]. For both pediatric and adult patients, inclusion requires initiation of CPR with chest compressions or defibrillation. Data collection follows the Utstein reporting recommendations and includes demographic data, arrest characteristics, treatment variables, response times, and outcomes [18].

At the time of the study, all Swedish hospitals with cardiac arrest response teams (*n* = 76) participated in the registry. Here, the term “complete Swedish cohort” refers to all pIHCA events reported to the SRCR during the study period, not necessarily all pIHCA events that occurred in Sweden. Because reporting completeness varied between hospitals, clinical settings, and calendar years, the cohort should be interpreted as a complete registry‐reported cohort [19]. Thirty‐two of the hospitals have pediatric clinics, many of which also offer in‐patient care. Hospitals providing exclusively elective care without emergency department services do not contribute to the in‐hospital section of the registry and were not represented in the study population. Patients with OHCA admitted to hospital with ongoing CPR are not included in the in‐hospital part of the SRCR; the same applies for newborn infants on maternity wards who have never had independent circulation.

### 2.3 Variables and Outcomes

The variables analyzed included age, sex, congenital heart disease, prematurity, location of arrest, defibrillation, time from arrest to alarm, time from arrest to CPR initiation, time from arrest to response team arrival, and time from arrest to ROSC. The primary outcome was survival at 30 days following pIHCA. Survival time was calculated from the date of cardiac arrest. Response‐time variables were analyzed among events with available data. Several variables had missing data, including congenital heart disease and prematurity. Missing values were not imputed. Therefore, percentages were calculated using the number of events with available data for each variable, and the denominators might therefore vary.

### 2.4 Statistical Analyses

Analyses were performed using the SAS software version 9.4 (SAS Institute, Cary, NC, USA). Continuous variables are presented as medians with interquartile ranges (IQR), and categorical variables are presented as counts and percentages. Percentages were calculated using the number of events with available data for each variable. Survival probabilities were estimated using the Kaplan–Meier method. Survival curves were stratified by sex, age group, and location of arrest. Differences between groups were assessed using log‐rank tests. *p*‐values below 0.05 were considered statistically significant. Due to the small number of events in several subgroups, further statistical analyses such as multivariable survival modelling were not performed. The survival analyses should therefore be interpreted as descriptive and exploratory.

### 2.5 Ethics Approval

The study was approved by the Swedish Ethical Review Authority, Lund Regional Office, Dnr 2025‐05759‐01.

## 3 Results

### 3.1 Study Population and Patient Characteristics

**FIGURE 1:**
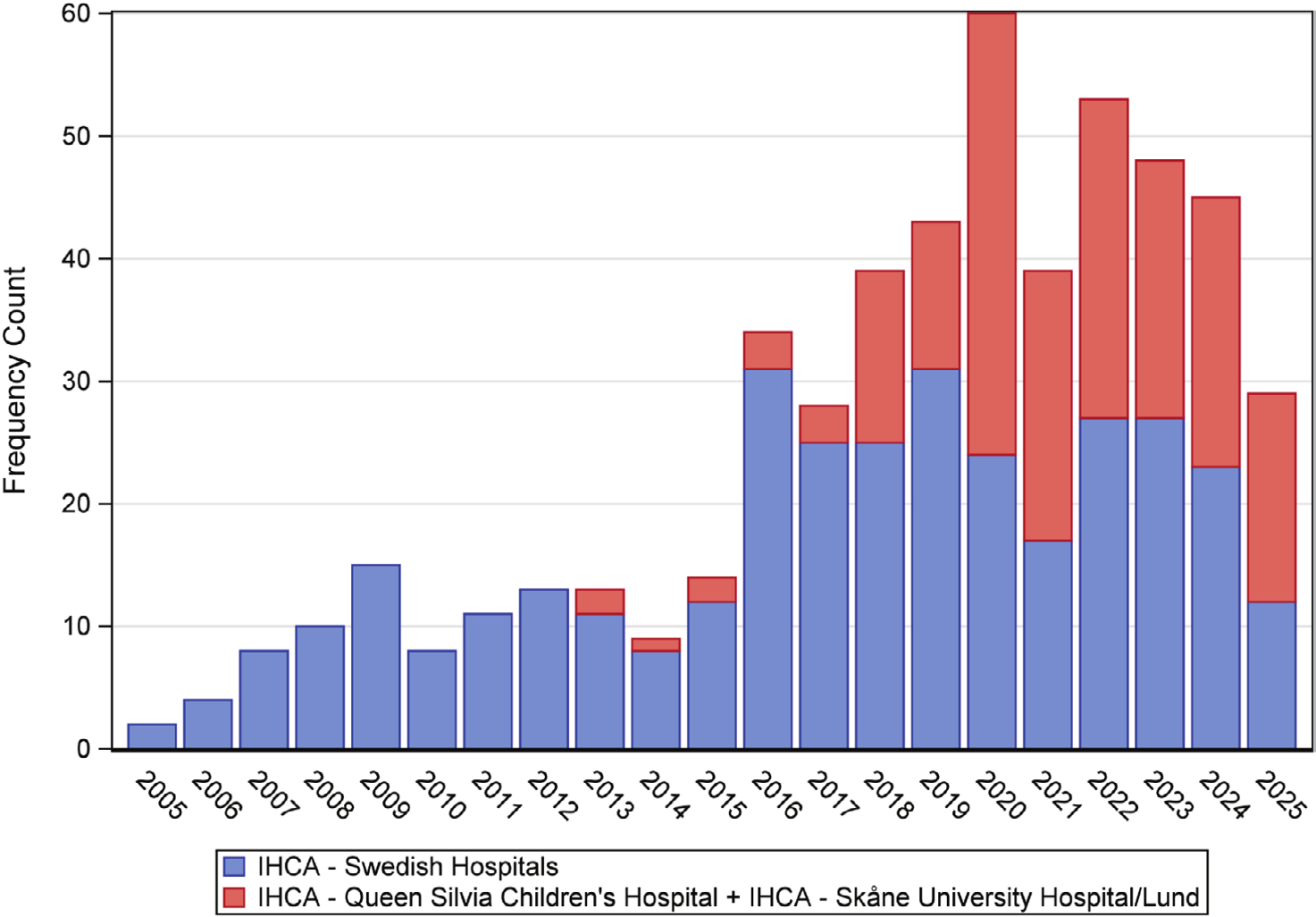
Number of pediatric in‐hospital cardiac arrests reported to the SRCR from 2005 to 2025. Events from the two national centers for highly specialized pediatric cardiology and cardiac surgery, Queen Silvia Children's Hospital and Skane University Hospital/Lund, are shown separately from those reported by other Swedish hospitals.

Between January 1, 2005 and September 25, 2025, 525 pIHCA events were reported to the SRCR. Of these, 344 events occurred at Swedish hospitals other than the two national centers for highly specialized pediatric cardiology and cardiac surgery, Queen Silvia Children's Hospital (DSBS) and Skåne University Hospital/Lund (SUS/L), while 181 events occurred at DSBS or SUS/L. Among the 344 events occurring outside DSBS and SUS/L, 242 (70.3%) were registered during 2016–2025, compared with 102 (29.7%) during 2005–2015. The number of registered events increased over the study period, with a particularly marked contribution from DSBS and SUS/L during the later years (Figure 1).

In the cohort treated outside DSBS and SUS/L, infants younger than 1 year constituted the largest age group (35.2%, *n* = 121), followed by children aged 1–5 years (25.6%, *n* = 88) and adolescents aged 16–18 years (19.2%, *n* = 66). Girls accounted for 45.3% (*n* = 156) of events. The patient population at DSBS and SUS/L differed substantially from that at the other Swedish hospitals. Infants accounted for 61.9% of arrests at the two highly specialized centers. Among patients with available data, congenital heart disease was reported in 55.1% (75/136) versus 14.4% (21/146), and prematurity in 30.5% (36/118) versus 13.1% (16/122), respectively (Table 1).

**TABLE 1:** Demographics and clinical characteristics of the cardiac arrests.

Variable	All Swedish hospitals (*n* = 525)	Excluding DSBS and SUS/L (*n* = 344)	DSBS and SUS/L (*n* = 181)
Period			
2005–2015	107 (20.4%)	102 (29.7%)	5 (2.8%)
2016–2025	418 (79.6%)	242 (70.3%)	176 (97.2%)
Age group in years, *n* (%)			
< 1	233 (44.4%)	121 (35.2%)	112 (61.9%)
1–5	126 (24.0%)	88 (25.6%)	38 (21.0%)
6–10	34 (6.5%)	25 (7.3%)	9 (5.0%)
11–15	55 (10.5%)	44 (12.8%)	11 (6.1%)
16–18	77 (14.7%)	66 (19.2%)	11 (6.1%)
Sex	*n* = 525	*n* = 344	*n* = 181
Girls	231 (44.0%)	156 (45.3%)	75 (41.4%)
Congenital heart disease	*n* = 282	*n* = 146	*n* = 136
Yes *n* (%)	96 (34.0%)	21 (14.4%)	75 (55.1%)
Prematurity	*n* = 240	*n* = 122	*n* = 118
Yes *n* (%)	52 (21.7%)	16 (13.1%)	36 (30.5%)
Arrest characteristics			
Location of cardiac arrest, *n* (%)	*n* = 525	*n* = 344	*n* = 181
Ward	147 (28.0%)	109 (31.7%)	38 (21.0%)
ICU	168 (32.0%)	78 (22.7%)	90 (49.7%)
Emergency room	98 (18.7%)	90 (26.2%)	8 (4.4%)
Operating room	56 (10.7%)	23 (6.7%)	33 (18.2%)
Other	56 (10.7%)	44 (12.8%)	12 (6.6%)
Defibrillation performed, *n* (%)	*n* = 525	*n* = 344	*n* = 181
No	365 (69.5%)	232 (67.4%)	133 (73.5%)
Yes	43 (8.2%)	31 (9.0%)	12 (6.6%)
Missing	117 (22.3%)	81 (23.5%)	36 (19.9%)

*Note:* For categorical variables, *n* (%) is presented.

Abbreviations: DSBS = Queen Silvia Children's Hospital, SUS/L = Skåne University Hospital/Lund.

### 3.2 Arrest Characteristics

Among the 344 pIHCA events occurring outside DSBS and SUS/L, the most common arrest location was a hospital ward (31.7%, *n* = 109), followed by the emergency department (26.2%, *n* = 90) and the intensive care unit (ICU; 22.7%, *n* = 78). Cardiac arrests in the operating room accounted for 6.7% (*n* = 23), while 12.8% (*n* = 44) occurred at other hospital locations.

The distribution of arrest locations differed markedly at DSBS and SUS/L, where 49.7% of arrests occurred in the ICU and 18.2% in the operating room. Conversely, emergency department arrests accounted for 26.2% of events outside DSBS and SUS/L but only 4.4% at the two highly specialized centers (Table 1).

### 3.3 Response Time

Among events outside DSBS and SUS/L with available response‐time data, the median time from cardiac arrest to CPR initiation was 0 min (IQR 0;0), with CPR initiated immediately in 81.4% (237/291) and within five minutes in 99.3% of events. Median time from arrest to response‐team arrival was 3 min (IQR 1;5), and median time from arrest to ROSC was 5 min (IQR 2–16). Response times were broadly similar between the two groups (Table 2).

**TABLE 2:** Response times: time from cardiac arrest to events in the resuscitation process.

Response time variables in minutes	All Swedish hospitals (*n* = 525)	Excluding DSBS and SUS/L (*n* = 344)	DSBS and SUS/L (*n* = 181)
Arrest to alarm, median (IQR)^a^	*n* = 263	*n* = 201	*n* = 62
Categorical, *n* (%)	0 (0; 1)	0 (0; 1)	0 (0; 1)
< 0	34 (11.4%)	27 (11.8%)	7 (10.1%)
0	145 (48.8%)	106 (46.5%)	39 (56.5%)
1–5	63 (21.2%)	52 (22.8%)	11 (15.9%)
6–10	26 (8.8%)	21 (9.2%)	5 (7.2%)
≥ 11	29 (9.8%)	22 (9.6%)	7 (10.1%)
Arrest to CPR initiation, median (IQR)	*n* = 468	*n* = 291	*n* = 177
Categorical, *n* (%)	0 (0; 0)	0 (0; 0)	0 (0; 0)
0	386 (82.5%)	237 (81.4%)	149 (84.2%)
1–5	78 (16.7%)	52 (17.9%)	26 (14.7%)
6–10	2 (0.4%)	1 (0.3%)	1 (0.6%)
≥ 11	2 (0.4%)	1 (0.3%)	1 (0.6%)
Arrest to team arrival, median (IQR)^a^	*n* = 235	*n* = 181	*n* = 54
Categorical, *n* (%)	3 (1; 5)	3 (1; 5)	3 (2; 5)
< 0	29 (11.0%)	23 (11.3%)	6 (10.0%)
0	35 (13.3%)	25 (12.3%)	10 (16.7%)
1–5	159 (60.2%)	123 (60.3%)	36 (60.0%)
6–10	32 (12.1%)	25 (12.3%)	7 (11.7%)
≥ 11	9 (3.4%)	8 (3.9%)	1 (1.7%)
Arrest to ROSC, median (IQR)	*n* = 240	*n* = 108	*n* = 132
Categorical, *n* (%)	4 (2; 12.5)	5 (2; 16)	3.5 (2; 10)
< 0	1 (0.4%)	1 (0.9%)	0 (0.0%)
0	13 (5.4%)	2 (1.8%)	11 (8.3%)
1–5	129 (53.5%)	56 (51.4%)	73 (55.3%)
6–10	29 (12.0%)	12 (11.0%)	17 (12.9%)
≥ 11	69 (28.6%)	38 (34.9%)	31 (23.5%)

Abbreviations: CPR = cardiopulmonary resuscitation; DSBS = Queen Silvia Children's Hospital; IQR = interquartile range; ROSC = return of spontaneous circulation; SUS/L = Skåne University Hospital/Lund.

^a^ Excluding negative values.

### 3.4 Survival

**FIGURE 2:**
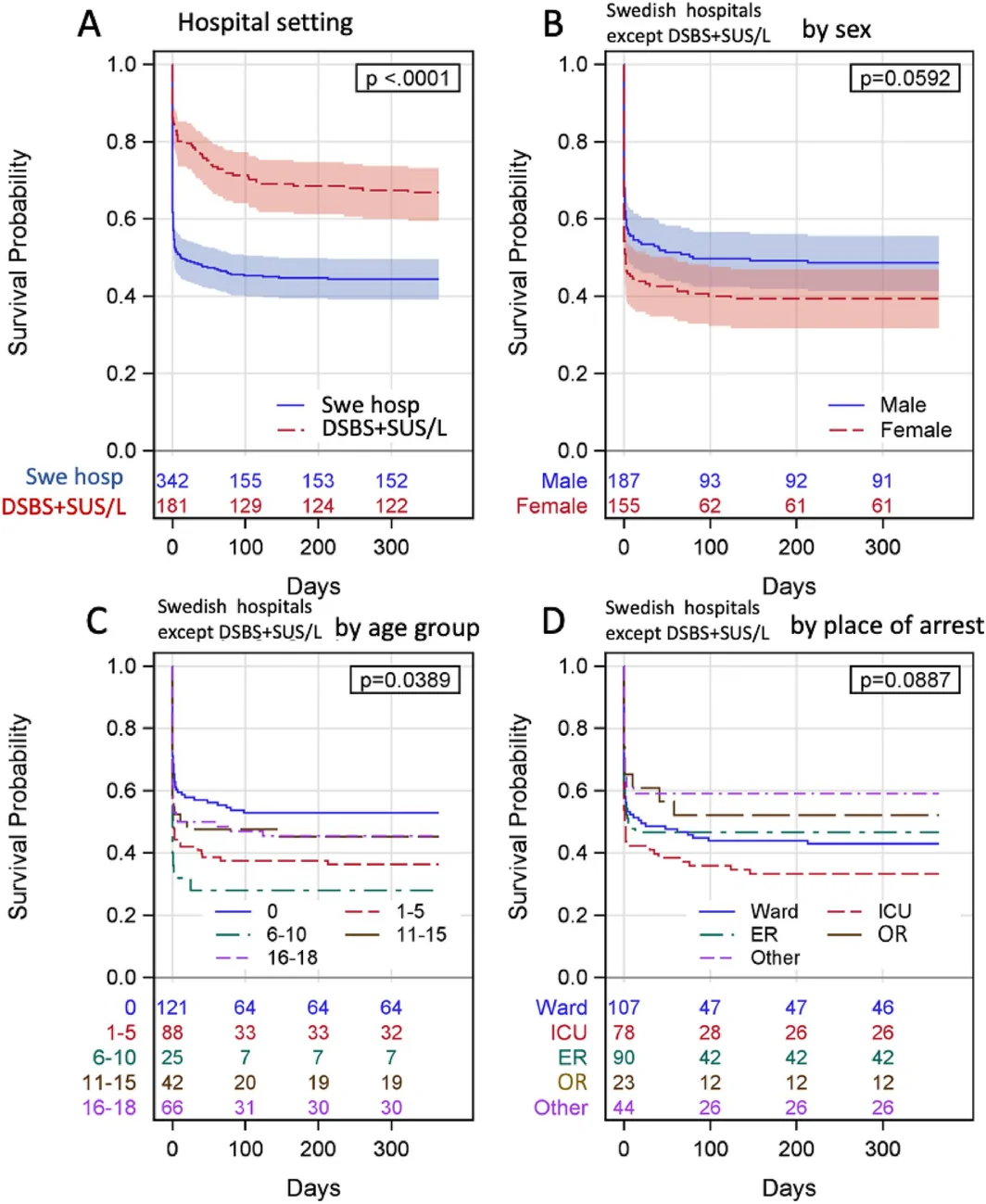
Unadjusted Kaplan–Meier analyses. One year survival. (A) Survival at Swedish hospitals outside DSBS and SUS/L compared with survival at DSBS and SUS/L. Swedish hospitals outside DSBS and SUS/L survival by (B) gender, (C) age group, and (D) place of arrest. DSBS = Queen Silvia Children's Hospital; ER = emergency room; ICU = intensive care unit; OR = operating room; SUS/L = Skåne University Hospital/Lund.

Among children with pIHCA at Swedish hospitals other than DSBS and SUS/L, ROSC was achieved in 62%, 49% survived to 30 days, and 44% survived to 1 year (Table 3). Survival differed across age groups in unadjusted Kaplan–Meier analysis (log‐rank *p* = 0.0389; Figure 2C). Infants had the highest observed survival outside DSBS and SUS/L, with ROSC achieved in 71%, 30‐day survival of 57%, and 1‐year survival of 53%. The lowest 30‐day survival was observed among children aged 6–10 years (28%). Thirty‐day survival was 42% among children aged 1–5 years, 48% among those aged 11–15 years, and 50% among adolescents aged 16–18 years. Observed outcomes also varied by arrest location. Among events outside DSBS and SUS/L, 30‐day survival was highest for arrests occurring in the operating room (61%), followed by other hospital locations (59%), hospital wards (49%), emergency departments (47%), and ICUs (41%). Corresponding 1‐year survival was 52%, 59%, 43%, 47%, and 33%, respectively. Differences in survival by arrest location did not reach statistical significance in the unadjusted Kaplan–Meier analysis (log‐rank *p* = 0.0887; Figure 2D). No statistically significant difference in survival was observed between boys and girls (log‐rank *p* = 0.0592; Figure 2B). Thirty‐day survival was 53% among boys and 43% among girls, with corresponding 1‐year survival of 49% and 39%, respectively.

**TABLE 3:** Survival: ROSC, 30 days, and 1 year.

Variable		All Swedish hospitals			Excluding DSBS and SUS/L			DSBS and SUS/L		
		ROSC	30 days	1 year	ROSC	30 days	1 year	ROSC	30 days	1 year
Total		0.64	0.52	0.47	0.62	0.49	0.44	0.77	0.70	0.64
Sex	Boys	0.69	0.55	0.50	0.68	0.53	0.49	0.74	0.65	0.61
	Girls	0.58	0.47	0.43	0.54	0.43	0.39	0.80	0.76	0.68
Age	0	0.74	0.63	0.58	0.71	0.57	0.53	0.87	0.87	0.77
	1–5	0.60	0.45	0.40	0.57	0.42	0.36	0.83	0.67	0.67
	6–10	0.41	0.26	0.26	0.40	0.28	0.28	0.50	0.00	0.00
	11–15	0.60	0.51	0.49	0.57	0.48	0.45	0.80	0.80	0.80
	16–18	0.59	0.47	0.42	0.62	0.50	0.45	0.29	0.14	0.14
Place of arrest	Ward	0.62	0.48	0.42	0.63	0.49	0.43	0.55	0.45	0.27
	ICU	0.60	0.48	0.41	0.54	0.41	0.33	0.84	0.79	0.74
	Emergency room	0.60	0.47	0.47	0.60	0.47	0.47	0.50	0.50	0.50
	Operating room	0.81	0.72	0.67	0.74	0.61	0.52	0.92	0.92	0.92
	Other	0.72	0.58	0.58	0.70	0.59	0.59	0.78	0.56	0.56

Abbreviations: DSBS = Queen Silvia Children's Hospital; ROSC = return of spontaneous circulation; SUS/L = Skåne University Hospital/Lund.

Outcomes were more favorable at DSBS and SUS/L than at the other Swedish hospitals. At the two highly specialized centers, ROSC was achieved in 77% of events, 30‐day survival was 70%, and 1‐year survival was 64%, compared with 62%, 49%, and 44%, respectively, at other Swedish hospitals. Overall survival differed significantly between the two groups in unadjusted Kaplan–Meier analysis (log‐rank *p* < 0.0001; Figure 2A).

## 4 Discussion

In this national registry‐based study, we describe pIHCA reported from Swedish hospitals over a 20‐year period, with particular focus on the population treated outside the two national centers for highly specialized pediatric cardiology and cardiac surgery, Queen Silvia Children's hospital (DSBS) and Skåne University Hospital/Lund (SUS/L). Arrest location, patient characteristics, and survival differed substantially between the broader Swedish hospital population and DSBS and SUS/L.

Relatively few cases were reported before 2016, accounting for 29.7% of events in the broader Swedish hospital population and only 2.8% of events at DSBS and SUS/L. The number of registered events has increased substantially over time; however, this should not be interpreted as an increase in the incidence of pIHCA. Rather, the increase likely reflects changes in registry participation and reporting, including the introduction of pediatric‐specific variables in 2018 [15]. Consequently, the present study describes reported pIHCA events rather than the national incidence or temporal trends.

The distribution of pIHCA across hospital locations was notably different between the two populations. Outside DSBS and SUS/L, 22.7% of arrests occurred in an ICU, 31.7% on hospital wards, and 26.2% in emergency departments. This differs from national and international pIHCA cohorts, in which ICUs account for a large proportion of reported arrests [13, 16, 20]. A similar pattern was observed within the highly specialized centers in the present study, where almost half of the arrests occurred in the ICU (49.7%) and a further 18.2% in the operating room. Thus, studies based predominantly on specialized pediatric centers may not fully reflect the distribution of pIHCA across a broader national hospital system.

Marked differences in patient characteristics further illustrate the differences between these hospital populations. At DSBS and SUS/L, 61.9% of arrests occurred in infants compared with 35.2% at other Swedish hospitals. Among patients with available data, congenital heart disease was reported in 55.1% compared with 14.4%, and prematurity, 30.5% compared with 13.1%, respectively. These differences are not unexpected, given that DSBS and SUS/L are national referral centers for highly specialized pediatric cardiac care and therefore treat a large population of children with congenital heart disease. Nevertheless, the findings demonstrate that pIHCA is not a homogenous clinical entity and emphasize the importance of considering hospital type and case mix when interpreting national and international pIHCA data.

Among Swedish hospitals outside DSBS and SUS/L, 30‐day survival was 49% and 1‐year survival 44%. When all Swedish hospitals were considered together, 30‐day survival was 52%, which is in line with contemporary pIHCA reports [21]. However, survival was higher at DSBS and SUS/L, with 70% surviving to 30 days and 64% to 1year, and the unadjusted Kaplan–Meier analysis also demonstrated a significant difference in survival between the hospital groups (*p* < 0.0001). However, these findings should not be used as benchmark estimates for hospital performance or quality of care. The study was not designed for hospital‐level comparisons, and the groups may differ in patient selection, underlying disease, arrest location, monitoring level, and clinical setting, while differences in reporting completeness and missing data may also have influenced the results. We report ROSC, 30‐day survival, and 1‐year survival because these outcomes represent different stages of the postarrest clinical course. However, survival alone does not capture neurological or functional recovery, which remains an important limitation in pediatric cardiac arrest research [22].

The highest observed ROSC rate was seen among children with cardiac arrest in the operating room. This is clinically plausible, as these patients are continuously monitored with immediate access to personnel trained in resuscitation. Perioperative arrests may result from rapidly identifiable and potentially reversible causes.

Interestingly, response times were similar across Swedish hospitals, with a median time from arrest to CPR initiation of 0 and 3 min to response‐team arrival. More than 80% received CPR immediately, likely reflecting that many pIHCA are witnessed or occur in closely monitored settings. It also indicates rapid recognition and initiation of resuscitation across Swedish hospitals and is consistent with current recommendations for IHCA [23]. As pediatric cardiac arrest is often preceded by clinical deterioration rather than a sudden arrhythmic event, early recognition and prompt intervention may contribute to the relatively favorable survival observed in this cohort.

The substantial differences in survival despite similar response times suggest that other factors including prearrest condition, arrest etiology, illness severity, monitoring, and availability of advanced treatment may be important determinants of outcome.

### 4.1 Strengths and Limitations

Several registry‐related factors should be considered when interpreting the findings. Although the study is national in scope, the data represent pIHCA events reported to the SRCR and may not include all pIHCA events occurring in Sweden. The cohort should therefore be interpreted as a national registry‐reported cohort.

Because reporting completeness varied between hospitals, clinical settings and calendar year, as well as key variables demonstrated missing data, the findings should be interpreted as descriptive and exploratory rather than as benchmark data for hospital performance. Missing data were particularly important for congenital heart disease (available in 282/525 events) and prematurity (240/525), limiting the ability to fully compare the case mix and risk profile between DSBS, SUS/L, and other Swedish hospitals. The registry also lacked detailed information on prearrest physiology, arrest etiology, illness severity, and neurological outcome. These factors are important for understanding why cardiac arrest occurs and why outcomes differ between patients and hospital settings. Consequently, differences in case mix could not be fully accounted for when interpreting the observed differences in ROSC and survival between hospital groups.

Despite these limitations, this study provides important information on pIHCA across a broad range of Swedish hospital settings during an extended period of time. By examining the broader Swedish hospital population separately from the two national highly specialized pediatric cardiac centers, the study also illustrates how hospital type and case mix may influence the characteristics and outcomes of a national pIHCA cohort.

Future studies should aim to improve the completeness of pIHCA reporting across Swedish hospitals, particularly in pediatric intensive care and referral centers. More detailed information on prearrest physiology, arrest etiology, illness severity, monitoring, and neurological outcome would enable a more comprehensive understanding of pIHCA and facilitate more meaningful comparisons across hospital settings [24].

## 5 Conclusion

pIHCA reported from Swedish hospitals outside the two national centers for highly specialized pediatric cardiac care was associated with 49% 30‐day survival and 44% 1‐year survival. Patient characteristics, arrest location, and survival differed substantially from those at the two highly specialized centers, highlighting the heterogeneity of pIHCA across hospital settings. These findings underscore the importance of considering hospital type and case mix when interpreting pIHCA data.

## Author Contributions

**Hannah Fovaeus:** conceptualization, data curation, formal analysis, investigation, methodology, writing‐original draft, writing‐reviewing and editing. **Johan Holmen:** conceptualization, investigation, methodology, writing‐reviewing and editing. **Zacharias Mandalenakis:** methodology, writing‐reviewing and editing. **Matilda Frisk Torell:** conceptualization, methodology, writing‐reviewing and editing. **Mattias Molin:** data curation, formal analysis, and methodology. **Araz Rawshani:** conceptualization, methodology, writing‐reviewing and editing. **Albert Gyllencreutz Castellheim:** conceptualization, data curation, formal analysis, methodology, supervision, writing‐reviewing and editing.

## Funding

The authors have nothing to report.

## Conflicts of Interest

The authors declare no conflicts of interest.

## Data Availability

The data that support the findings of this study are available on request from the corresponding author. The data are not publicly available due to privacy or ethical restrictions.
